# Protocol for a randomised pilot multiple centre trial of conservative versus liberal oxygenation targets in critically ill children (Oxy-PICU)

**DOI:** 10.1136/bmjopen-2017-019253

**Published:** 2017-12-14

**Authors:** Gareth A L Jones, Padmanabhan Ramnarayan, Sainath Raman, David Inwald, Michael P W Grocott, Simon Eaton, Samiran Ray, Michael J Griksaitis, John Pappachan, Daisy Wiley, Paul R Mouncey, Jerome Wulff, David A Harrison, Kathryn M Rowan, Mark J Peters

**Affiliations:** 1 Respiratory Critical Care and Anaesthesia Unit, UCL Great Ormond Street Institute of Child Health, London, UK; 2 Paediatric Intensive Care Unit, Great Ormond Street Hospital, London, UK; 3 Children’s Acute Transport Service, Great Ormond Street Hospital, London, UK; 4 Paediatric Intensive Care Unit, St Mary’s Hospital, Imperial College Healthcare NHS Trust, London, UK; 5 Critical Care Research Group, Southampton NIHR Biomedical Research Centre, University Hospital Southampton NHS Foundation Trust/University of Southampton, Southampton, UK; 6 Stem Cells and Regenerative Medicine Section, UCL Great Ormond Street Institute of Child Health, London, UK; 7 Paediatric Intensive Care Unit, Southampton General Hospital, University Hospital Southampton NHS Foundation Trust, Southampton, UK; 12 Clinical Trials Unit, Intensive Care National Audit & Research Centre, 24 High Holborn, London, UK

**Keywords:** child, critical care, hypoxia, hyperoxia

## Abstract

**Introduction:**

Optimal targets for systemic oxygenation in paediatric critical illness are unknown. Observational data indicate that high levels of arterial oxygenation are associated with poor outcomes in resuscitation of the newborn and in adult critical illness. Within paediatric intensive care units (PICUs), staff prevent severe hypoxia wherever possible, but beyond this there is no consensus. Practice varies widely with age, diagnosis, treating doctor and local or national guidelines followed, though peripheral blood oxygen saturations (SpO_2_) of >95% are often targeted. The overall aim of this pilot study is to determine the feasibility of performing a randomised trial in critically ill children comparing current practice of liberal SpO_2_ targets with a more conservative target.

**Methods and analysis:**

Oxy-PICU is a pragmatic, open, pilot randomised controlled trial in infants and children requiring mechanical ventilation and receiving supplemental oxygen for abnormal gas exchange accepted for emergency admission to one of three participating UK PICUs. The study groups will be either a conservative SpO_2_ target of 88%–92% (inclusive) or a liberal SpO_2_ target of >94%. Infants and children who fulfil all inclusion criteria and none of the exclusion criteria will be randomised 1:1 by a secure web-based system to one of the two groups. Baseline demographics and clinical status will be recorded as well as daily measures of oxygenation and organ support. Discharge outcomes will also be recorded. In addition to observational data, blood and urine samples will be taken to identify biochemical markers of oxidative stress. Outcomes are targeted at assessing study feasibility with a primary outcome of adequate study recruitment (target: 120 participants).

**Ethics and dissemination:**

The trial received Health Research Authority approval on 1 June 2017 (16/SC/0617). Study findings will be disseminated in national and international conferences and peer-reviewed journals.

**Trial registration number:**

NCT03040570.

**Strengths and limitations of this study**

- This is the first randomised trial to compare conservative and liberal systemic oxygenation targets in mechanically ventilated children.
- As this is a pilot study, without a formal power calculation, outcomes of clinical significance should be treated as hypothesis-generating only.
- If deemed to be feasible, the outcomes and evaluation of processes of this study will inform a larger appropriately powered definitive study.
- The pragmatic design of this study will allow us to assess the ability of clinical staff to adhere to the target treatment groups.

## Introduction

The optimal targets for systemic oxygenation in paediatric critical illness are unknown. Intensive care staff prevent severe hypoxia wherever possible, but beyond this there is no consensus. Practice varies widely with age, diagnosis and treating doctor.^1 2^ This uncertainty about the optimal oxygenation target is illustrated by the variance in national guidelines in even the most common cause of acute infant respiratory distress: respiratory syncytial virus bronchiolitis. The US American Academy of Pediatrics recommends a peripheral blood oxygen saturation (SpO_2_) target >90%, whereas the Scottish Intercollegiate Guidelines Network recommend SpO_2_ >94%.^3^

Oxygenation targets have become a ‘hot topic’ for clinical trials because of observational data suggesting that high levels of arterial oxygenation are associated with poor outcomes in resuscitation of the newborn, adult critical illness, myocardial infarction, postcardiac arrest and possibly also in respiratory failure.^4^ When added to the known risks of severe hypoxia, a ‘U-shaped’ relationship between arterial oxygenation and risk of death emerges.^5 6^ We have recently completed a systematic review of the paediatric literature addressing this issue, and although the data are scarce, the same pattern of increased risk and both high and low levels of arterial oxygenation has been observed.^7^ Our recent cohort study of 7410 critically ill children also demonstrated a ‘U-shaped’ relationship between admission arterial oxygen tension and survival. This pattern persists after adjustment for case-mix (including congenital cyanotic heart disease) and indicators of physiological severity. This complex relationship between oxygenation and outcome may reflect the balance between harm from hypoxia at one end of the oxygenation spectrum (low SpO_2_) and a combination of increased oxygen free radical damage and iatrogenic injury from more aggressive treatments at the other (high SpO_2_).^6^

The lack of evidence indicating the safest oxygen level for a critically ill child needs investigation. Around 19 000 such children are admitted to paediatric intensive care units (PICUs) in the UK annually. Around 75% receive artificial support for ventilation in some form. The primary aim of this artificial ventilation is to support blood oxygen values at a safe level. However, since the optimal level is unknown, clinicians typically default to targeting physiologically normal or even supranormal values. With other variables during critical illness, this approach of ‘normalisation of physiology’ is known to be either harmful or of no benefit.^8–11^ In neonatal and critically ill adult patients, the choice of oxygen targets are known to influence survival rates, lengths of stay and costs. Three large randomised studies in extreme preterm infants compared lower (85%–89%) with higher oxygen targets (91%–95%).^12^ Unexpectedly, an increased risk of death (1.45; 95% CI 1.15 to 1.84; P=0.002) was seen with the lower oxygen targets. In contrast, recent pilot data in adult critical illness demonstrate a trend towards the opposite effect: that is, reduced mortality with lower oxygenation targets in the sickest patients: relative risk 0.49 (95% CI 0.20 to 1.17; P=0.10).^13^ Similarly, harmful effects of supplemental oxygen have been demonstrated in adults with ST elevation myocardial infarction.^14 15^ In October 2016, a single-centre study in adults was stopped early because of a significant survival advantage: absolute risk reduction for intensive care unit mortality (8.6%, 1.7%–15% P=0.01) in the conservative oxygenation group.^16^ Even prior to these data, there has been a move in adult intensive care towards lower oxygen targets, for example, SpO_2_ 88%–95%.^4^

The only paediatric trial data—in non-critically ill children with bronchiolitis—demonstrate equivalent safety of a peripheral oxygen saturation (SpO_2_) target of >90% when compared with >94%. Later hospital discharges were seen with the higher target (ratio of length of stay: 1.28, 95% CI 1.09 to 1.50, P=0.003).^17^

Current practice favours very liberal oxygen targets. We have conducted an analysis of around 7 million SpO_2_ values averaged over 5 s in a single PICU: 30% of recorded values were 100% and >60% were >95%.^18^

### Study aim

The overall aim of this study is to determine if it is feasible to perform a safe, adequately powered and affordable definitive randomised controlled trial in critically ill children comparing current practice of liberal targets for systemic oxygen levels with more conservative targets. The underlying hypothesis of a definitive trial is that the harm of interventions to raise arterial oxygen saturation to >94% exceeds the benefits of these interventions.

### Study objectives

- To test the willingness of clinicians to screen, recruit and randomise eligible patients.
- To estimate the recruitment rate.
- To test, following randomisation, delivery of and adherence to, the intervention and demonstrate separation between the groups.
- To test acceptability of the deferred consenting procedures and participant information.
- To test follow-up for the identified, potential, patient-centred primary and other important secondary outcome measures and for adverse event (AE) reporting.
- To inform final selection of a patient-centred primary outcome measure.
- To estimate the characteristics (eg, SD) of the selected patient-centred primary outcome measure to inform sample size estimation.
- To inform content and time needed for final data collection.
- To investigate the feasibility of collecting samples suitable for estimation of ischaemic/oxidative injury and antioxidant status.

## Methods and analysis

### Study design and setting

Oxy-PICU is a pragmatic, open, pilot randomised controlled trial in infants and children accepted for emergency admission to participating PICUs. Study recruitment will be at three PICUs and their local PICU transport teams representing typical configurations for UK PICUs (general or combined ICUs in general academic medical centres or within stand-alone children’s hospitals).

### Trial population and eligibility criteria

Infants and children receiving treatment at a participating site who fulfil all of the inclusion criteria and none of the exclusion criteria below:

#### Inclusion criteria

- Less than 16 years and more than 38 weeks’ corrected gestational age.
- Emergency admission accepted to a PICU requiring mechanical ventilation within first 6 hours of face-to-face contact with PICU staff or transport team.
- Receiving supplemental oxygen for abnormal gas exchange.

#### Exclusion criteria

- Death perceived as imminent.
- Brain pathology/injury as primary reason for admission (eg, traumatic brain injury, post-cardiac arrest, stroke and convulsive status epilepticus without aspiration).
- Known pulmonary hypertension.
- Known or suspected sickle cell disease.
- Known or suspected uncorrected congenital cardiac disease.
- End-of-life care plan in place with limitation of resuscitation.
- Receiving long-term mechanical ventilation prior to this admission (non-invasive ventilation (NIV) or invasive ventilation (IV)).
- Recruited to Oxy-PICU in a previous admission.

### Screening and randomisation

**Figure 1:**
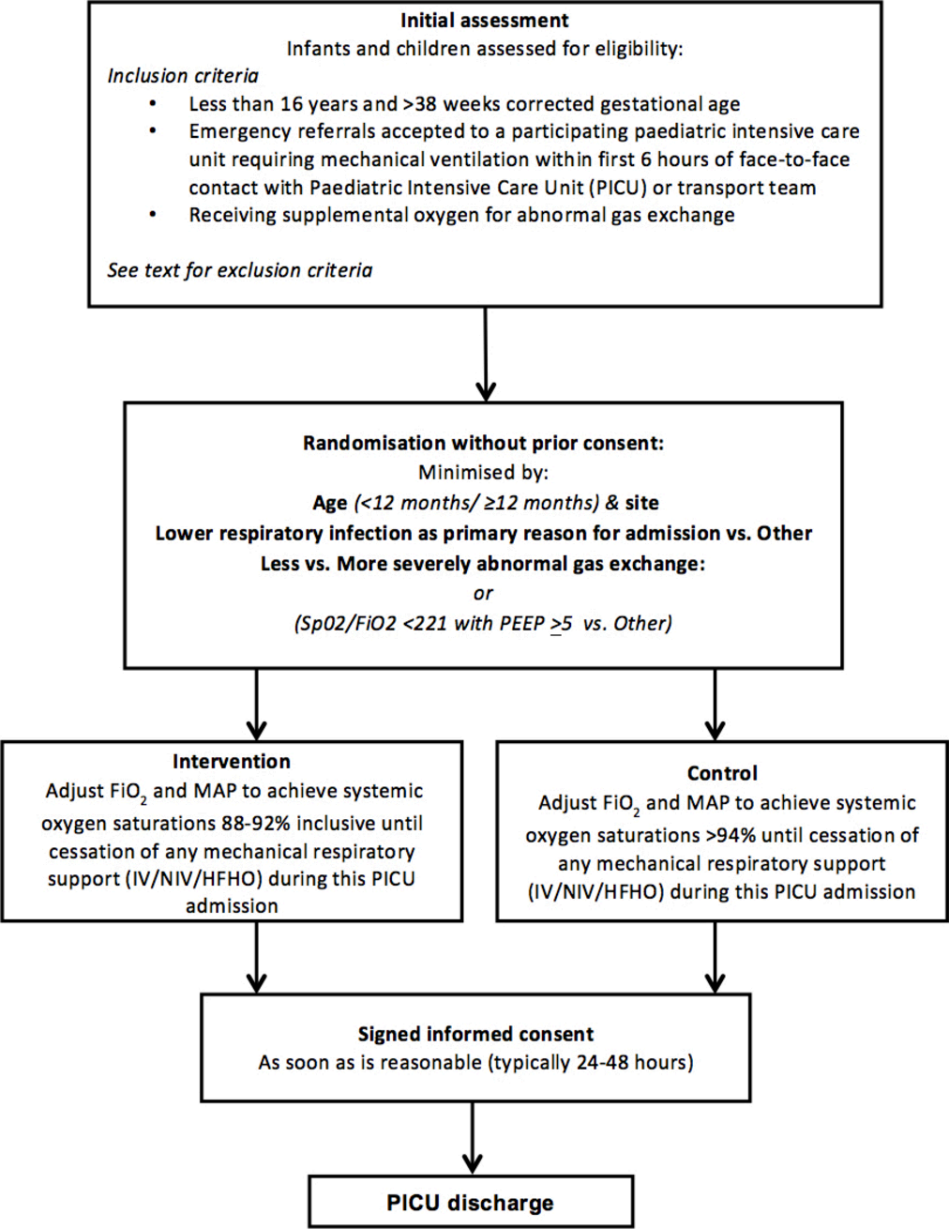
Trial schema of eligibility, randomisation and study intervention to discharge from PICU. FiO_2_, fraction of inspired oxygen; HFNO, high flow humidified oxygen; IV, invasive ventilation; MAP, mean airway pressure; NIV, non-invasive ventilation; PEEP, positive end expiratory pressure; PICU, paediatric intensive care unit; SpO_2,_ peripheral oxygen saturations.

Potentially eligible infants and children will be screened against the inclusion/exclusion criteria by transport team and PICU staff supported by the Oxy-PICU trial team (figure 1). Randomisation will then follow a ‘research without prior consent’ model. Randomisation will occur as soon as eligibility has been confirmed with the aim of commencing treatment as soon as possible within the first 6 hours of the infant or child being in face-to-face contact with the PICU or transport staff. Participants will be randomly allocated (1:1) to either the conservative (88%–92%) or liberal (>94%) oxygen target group by a computer-generated dynamic procedure (minimisation) with a random component. Minimisation will be performed on: age (<12 months/≥12 months), study site and primary reason for admission (lower respiratory tract infection vs other); and degree of abnormality of gas exchange: Saturation/FiO_2_ (S/F) ratio <221 with positive end expiratory pressure >5 versus other. Randomisation will be via a secure web-based system.

### Prerandomisation and postrandomisation care

Prior to randomisation, all care will be determined by the clinical team primarily responsible for the child’s treatment and care. Once randomised, all care other than that intended to achieve trial target SpO_2_ will be determined by the clinical team.

### Consent procedures

Patients receiving mechanical support to ventilation requiring supplemental oxygen will most often need oxygen treatment started in a life-threatening emergency, where any delay in commencing treatment will be detrimental. This will make any attempt to obtain fully informed consent from parents/legal representatives during an emergency inappropriate and cause additional anxiety to families who are already distressed by their child’s illness. Consent will therefore follow a deferred consent model following previous work and guidelines in this setting.^19 20^

Once notified of the recruitment of a patient to the study, a delegated member of the site research team will approach the parents/legal representatives as soon as practically and appropriately possible after randomisation to discuss the study and provide a participant information sheet (PIS) (usually within 24–48 hours of randomisation). If the participant has been discharged or has died prior to their parents/legal representatives being approached, then the parents/legal representatives will be approached by an appropriate team member at a later point and will be provided with a standard PIS or a specific PIS for bereaved parents/guardians (B-PIS).

A consent form will be provided including a statement that participation is voluntary and that consent can be withdrawn at any time without consequence (online supplementary material 1). Parents/legal representatives will be allowed time to read the PIS (or B-PIS) and have an opportunity to ask any questions they may have about their child’s participation in Oxy-PICU. Deferred consent can be sought from parents/legal representatives following the death of their child and prior to their departure from the hospital if appropriate.

10.1136/bmjopen-2017-019253.supp1 Supplementary file 1

For participants who are discharged alive from PICU but have not been consented prior to discharge from the hospital, a study team member will call their parents/legal representative within five working days of discharge. For all participants discharged prior to consent, their parents/legal representatives will subsequently be sent a covering letter, personalised by the most appropriate clinical team member, and a copy of the relevant PIS and consent form by post 4 weeks after randomisation. If there is no response after 4 weeks of sending the initial letter, a follow-up letter along with the appropriate PIS will be sent to the family. Both letters will explain how to opt-out of the study and provide telephone contact details if parents/legal representatives wish to discuss the study with a member of the site research team. The second letter will provide the same information as the first letter. In addition, this letter will also confirm that if no consent form is received within 4 weeks of receipt of the letter, then the participant’s data (samples collected will be destroyed) will be included in the study unless the family notify the site research team otherwise.^19^

Parents/legal representatives can refuse to give consent (non-consent) or withdraw from Oxy-PICU at any time during the study. All data collected up to the point of withdrawal will be retained and included in the study analysis. In order to monitor non-consent, a minimal dataset will be collected for each parent/legal representative approached but not consented. In the case of non-consent/withdrawal from the study, any samples collected will be destroyed.

### Consent questionnaire

For parents/legal representatives who are approached for consent in person, as part of the PIS, parents/legal representatives will be provided with information about the option to complete a questionnaire regarding their views on the consenting procedures for the Oxy-PICU pilot study (online supplementary material 2). Questionnaires will be used to highlight specific concerns related to the consent procedure or conduct of hospital staff and will be used to inform future research design.

10.1136/bmjopen-2017-019253.supp2 Supplementary file 2

### Trial intervention

#### Liberal group

Participants allocated to the liberal oxygenation group will receive supplemental oxygen and ventilator settings at the discretion of the treating clinical team with the aim of maintaining peripheral oxygen saturations >94%. This will be continued until all ventilator support (delivered invasively or non-invasively) has been discontinued during this PICU admission. All other care (including antimicrobial therapy, fluid therapy, analgesic and sedative agents and bronchodilator therapy) will be determined by the clinical team primarily responsible for the participant’s care.

#### Conservative group

Participants allocated to the conservative oxygenation group will receive supplemental oxygen and ventilator settings at the discretion of the treating clinical team with the aim of maintaining peripheral oxygen saturations between 88% and 92% (inclusive). This will be continued until all ventilator support (delivered invasively or non-invasively) has been discontinued during this PICU admission. All other care will be determined by the clinical team primarily responsible for the participant’s care.

### Data collection and follow-up

Detailed guidance for the collection of data will be provided in the trial-specific standard operating procedure. All data items will be objectively defined according to relevant national and international guidelines. Data will be collected at baseline prior to randomisation, daily and at discharge from PICU as detailed in table 1. Patients will be followed-up until discharge from a participating PICU. Data entered onto the secure trial database will undergo validation checks for completeness, accuracy and consistency of data. Queries on incomplete, inaccurate or inconsistent data will be sent to the research team at participating sites for resolution.

**Table 1:** Data collection time points for enrolled participants

Time point		
Baseline	Daily	PICU discharge
Age	Ventilation mode*	Date of discharge
Gender	Interventions for organ support†	Survival status
Weight	Hourly SpO_2_ values‡	Discharge diagnosis
Acute diagnosis	–	–
Chronic diagnosis	–	–
Severity of gas exchange	–	–
Cause of respiratory failure	–	–
PIM2r score	–	–

*Ventilation mode including FiO_2_ and mean airway pressure.

†Including use of vasoactive drugs, ECMO, blood transfusion, renal support and neuromuscular blocker or sedative drug infusions.

‡Hourly SpO_2_ values for first 24 hours, then 4 hourly until day 6 postrandomisation and 12 hourly thereafter until the end of mechanical ventilation.

ECMO, extracorporeal membrane oxygenation; FiO_2_, fraction of inspired oxygen; PICU, paediatric intensive care unit; PIM2r, paediatric index of mortality 2 recalibated; SpO_2_, peripheral oxygen saturations.

### Sample collection

Samples of blood and urine will be collected to assess for possible mechanisms of oxidative injury. Blood from indwelling invasive lines will be sampled within 24 hours of randomisation and again up to 72 hours postrandomisation (or immediately prior to removal of suitable invasive sampling lines in patients with an anticipated shorter length of stay). Plasma from samples of 1–1.5 mL of whole blood will be analysed for malondialdehyde, ischaemia-modified albumin and total antioxidant status. Leucocyte hypoxia-inducible factor-1 alpha mRNA expression will also be estimated. Other markers of plasma and leucocyte oxidative stress may be investigated. Urine samples will also be taken at the same time points (within 24 hours and up to 72 hours postrandomisation) to discover biomarkers of oxidative stress using metabolomics and proteomic analyses.

### Adverse events

All infants and children eligible for Oxy-PICU are critically ill and due to the complexity of their condition are at increased risk of experiencing AEs. Many of these events are expected as a result of the infant/child’s medical condition and standard treatment received in the PICU and may not be related to participation in the trial. Consequently, any AEs occurring as a result of the infant/child’s medical condition or standard critical care treatment will not be reported. Pre-existing conditions do not qualify as AEs unless they worsen but should be documented in the infant/child’s medical notes. AEs that occur between randomisation and PICU discharge must be recorded in the participant’s medical notes and on the Oxy-PICU case report form. Those meeting the definition of a serious AE (SAE) must, in addition, be recorded in the SAE log and reported to the Intensive Care National Audit & Research Centre (ICNARC) Clinical Trials Unit (CTU), using the trial specific Oxy-PICU SAE Reporting Form, by fax within 24 hours of observing or learning of the SAE.

### Trial monitoring and oversight

The ICNARC CTU will conduct at least one monitoring visit to participating sites during the course of the pilot study. The trial will be supervised by the Trial Steering Committee (TSC), which will be chaired by an independent member and will include at least two additional independent members and a service user representative.

Safety will be monitored by the Data Monitoring and Ethics Committee (DMEC) throughout the trial period. All members of the DMEC will be independent of both the Oxy-PICU Trial Management Group and the TSC. The DMEC will operate under the DAMOCLES Charter^17 18^ and will report to the TSC, making recommendations on the continuation, or not, of the trial. The ICNARC CTU will be responsible for the day-to-day management of the trial and will act as custodian of the data. The ICNARC CTU will ensure that all SAEs are reported, as appropriate, to the REC.

### Outcome measures

As this is a pilot study outcome measures for the study will be focused on assessing the study feasibility for a larger scale definitive study as noted in box below.

Box **Outcome measures of the Oxy-PICU feasibility study**

**Primary outcome measure**

- Number of eligible patients recruited per site per month.

**Secondary outcome measures**

- Proportion of parents/legal representatives refusing deferred consent.
- Proportion of eligible patients randomised (target 50%).
- Distribution of time to randomisation.
- Proportion of systemic oxygen saturations within the target range in each group.
- Proportion of patients in each arm requiring other treatments influencing tissue oxygen delivery (blood transfusion and inotropic support).
- Characteristics and completeness of potential primary endpoints for a definitive study including: length of ventilation, length of paediatric intensive care unit (PICU) stay, PICU mortality and days of organ specific support.
- Observed adverse events.
- Time taken for data collection and entry.
- Distribution of markers of ischaemia and antioxidant status.

### Statistics

#### Power calculation

The Oxy-PICU pilot study is primarily set-up to test the feasibility of the protocol to recruit eligible patients. Therefore, there is no primary outcome to be compared between the two groups and, hence, a usual power calculation to determine sample size is not appropriate. Instead, the sample size has been determined to be adequate to estimate critical parameters to be tested to a necessary degree of precision. Based on available data from PICANet, it is anticipated that the participating sites will recruit approximately 4–10 children per month, providing a total of approximately 120 children in 6 months.

#### Statistical analysis

Descriptive analysis will be conducted to assess the objectives of Oxy-PICU. All analyses will be carried out on an intention-to-treat basis. Screening data will be reported by site and randomisation, and consent data will be reported by site and treatment group. Baseline demographic and clinical data as detailed in table 1 will be summarised by group and overall. Continuous variables will be summarised as mean (SD) and median (IQR) while categorical variables will be summarised as number (percentage).

Adherence to the intervention will be reported as:

- Time in SpO_2_ target range (hours) – mean (SD) and median (IQR) will be reported by treatment group.
- Percentage of time points SpO_2_ measurements in range by treatment group.

Daily organ support procedures will be presented graphically by plotting the percentage of patients in each group receiving each recorded support procedure. Daily interventions received will be presented graphically by plotting the mean (SD) of each intervention.

A comparison of outcomes by treatment group will be reported for the following outcome measures:

- Length of PICU stay.
- Length of IV.
- Length of NIV.
- Ventilator-free days at day 30.
- Duration of organ support.
- PICU mortality.

### Ethical compliance

The Oxy-PICU pilot study will be conducted in accordance with the approved trial protocol, ICH Good Clinical Practice (GCP) guidelines, the Data Protection Act (1998), the Mental Capacity Act (2005), as well as the ICNARC CTU’s research policies and procedures.

### Dissemination

Results of this pilot study will be presented at local, national and international conferences and also published in peer-reviewed journals.

### Participant confidentiality and data protection

No identifiable participant data will be required by the ICNARC CTU, as all follow-up data will be collected at participating sites. All participant data will be stored securely. ICNARC is registered under the Data Protection Act (1998), and all ICNARC CTU staff have undergone data protection and International Conference on Harmonisation (ICH) GCP training.

## Discussion

We believe that there is an urgent need for high-quality clinical evidence to inform on the optimal targets of systemic oxygenation during paediatric critical illness. Inferences ‘up’ from extremely premature infants or ‘down’ from adults are in conflict and unlikely to be valid in children because of age-related differences in the acute physiological responses to hypoxia/hyperoxia and distinct comorbidities.

A clinical trial comparing current (liberal) targets for systemic oxygenation with lower (conservative) targets in critically ill children is therefore required. This pilot study is a crucial step to understand if this is possible and also affords the opportunity to learn more about the biological mechanisms underlying costs and benefits of oxygen therapy in children.

## Supplementary Material

Reviewer comments

## References

[1] Raman S, Ray S, Peters MJ Survey of oxygen delivery practices in UK paediatric intensive care units. Crit Care Res Pract 2016;2016:1–4. 10.1155/2016/6312970 PMC4969506 27516901

[2] Helmerhorst HJ, Schultz MJ, van der Voort PH, et al Self-reported attitudes versus actual practice of oxygen therapy by ICU physicians and nurses. Ann Intensive Care 2014;4:23 10.1186/s13613-014-0023-y 25512878 PMC4240734

[3] Ralston SL, Lieberthal AS, Meissner HC, et al Clinical practice guideline: the diagnosis, management, and prevention of bronchiolitis. Pediatrics 2014;134:e1474–502. 10.1542/peds.2014-2742 25349312

[4] Hafner S, Beloncle F, Koch A, et al Hyperoxia in intensive care, emergency, and peri-operative medicine: Dr. Jekyll or Mr. Hyde? A 2015 update. Ann Intensive Care 2015;5:42 10.1186/s13613-015-0084-6 26585328 PMC4653126

[5] de Jonge E, Peelen L, Keijzers PJ, et al Association between administered oxygen, arterial partial oxygen pressure and mortality in mechanically ventilated intensive care unit patients. Crit Care 2008;12:R156 10.1186/cc7150 19077208 PMC2646321

[6] Martin DS, Grocott MP Oxygen therapy in critical illness: precise control of arterial oxygenation and permissive hypoxemia. Crit Care Med 2013;41:423–32. 10.1097/CCM.0b013e31826a44f6 23263574

[7] Raman S, Prince NJ, Hoskote A, et al Admission PaO2 and mortality in critically Ill children: a cohort study and systematic review. Pediatr Crit Care Med 2016;17:e444–50. 10.1097/PCC.0000000000000905 27509363

[8] Brower RG, Matthay MA, Morris A, et al Ventilation with lower tidal volumes as compared with traditional tidal volumes for acute lung injury and the acute respiratory distress syndrome. N Engl J Med 2000;342:1301–8. 10.1056/NEJM200005043421801 10793162

[9] Lacroix J, Hébert PC, Hutchison JS, et al Transfusion strategies for patients in pediatric intensive care units. N Engl J Med 2007;356:1609–19. 10.1056/NEJMoa066240 17442904

[10] Takala J, Ruokonen E, Webster NR, et al Increased mortality associated with growth hormone treatment in critically ill adults. N Engl J Med 1999;341:785–92. 10.1056/NEJM199909093411102 10477776

[11] Finfer S, Chittock DR, Su SY, et al Intensive versus conventional glucose control in critically ill patients. N Engl J Med 2009;360:1283–97. 10.1056/NEJMoa0810625 19318384

[12] Tarnow-Mordi W, Stenson B, Kirby A, et al Outcomes of two trials of oxygen-saturation targets in preterm infants. N Engl J Med 2016;374:749–60. 10.1056/NEJMoa1514212 26863265

[13] Panwar R, Hardie M, Bellomo R, et al Conservative versus liberal oxygenation targets for mechanically ventilated patients. A pilot multicenter randomized controlled trial. Am J Respir Crit Care Med 2016;193:43–51. 10.1164/rccm.201505-1019OC 26334785

[14] Stub D, Smith K, Bernard S, et al Air versus oxygen in ST-segment-elevation myocardial infarction. Circulation 2015;131:2143–50. 10.1161/CIRCULATIONAHA.114.014494 26002889

[15] Khoshnood A, Carlsson M, Akbarzadeh M, et al The effects of oxygen therapy on myocardial salvage in ST elevation myocardial infarction treated with acute percutaneous coronary intervention: the Supplemental Oxygen in Catheterized Coronary Emergency Reperfusion (SOCCER) Study. Cardiology 2015;132:16–21. 10.1159/000398786 25998033

[16] Girardis M, Busani S, Damiani E, et al Effect of conservative vs conventional oxygen therapy on mortality among patients in an intensive care unit: the oxygen-ICU randomized clinical trial. JAMA 2016;316:1583–9. 10.1001/jama.2016.11993 27706466

[17] Cunningham S, Rodriguez A, Boyd KA, et al Bronchiolitis of Infancy Discharge Study (BIDS): a multicentre, parallel-group, double-blind, randomised controlled, equivalence trial with economic evaluation. Health Technol Assess 2015;19:1–172. 10.3310/hta19710 PMC4780975 26364905

[18] Ray S, Rogers L, Raman S, et al Liberal oxygenation in paediatric intensive care: retrospective analysis of high-resolution _SpO2 data_. Intensive Care Med 2017;43:146–7. 10.1007/s00134-016-4606-y 27796402

[19] O’Hara CB, Canter RR, Mouncey PR, et al A qualitative feasibility study to inform a randomised controlled trial of fluid bolus therapy in septic shock. Arch Dis Child 2017 10.1136/archdischild-2016-312515 PMC5754873 28847877

[20] Woolfall K, Frith L, Dawson A, et al Fifteen-minute consultation: an evidence-based approach to research without prior consent (deferred consent) in neonatal and paediatric critical care trials. Arch Dis Child Educ Pract Ed 2016;101:49–53. 10.1136/archdischild-2015-309245 26464416 PMC4752644

