# Supplementary material for: Protocol for a randomised pilot multiple centre trial of conservative versus liberal oxygenation targets in critically ill children (Oxy-PICU)
Source: BMJ Open. 2017 Dec 14;7(12):e019253. doi: 10.1136/bmjopen-2017-019253 (PMC5736024; doi:10.1136/bmjopen-2017-019253)
Supplement: Supplementary file 1 [file bmjopen-2017-019253supp001.pdf]

icnarc intensive care national audit & research centre

IRAS Number: 212228

Oxy-PICU

CONSENT FORM

Parent / Guardian

Version 1.1, 1 December 2016

Site name: ______________________________ Trial Number: _________________

Child's name: ____

(Please write child's full name in block capitals here)

Please <u>initial</u> each line below

1. I confirm that I have read and understand the Participant Information Sheet (Parents/Legal Representative) (version 1.1 dated 1 December 2016) for the above study. I have had the opportunity to consider the information, ask questions and have had these answered satisfactorily. ___ ___ ___

2. I understand that the use of my child's information is voluntary, and that I am free to withdraw consent at any time, without giving any reason. ___ ___ ___

3. I understand that relevant sections of my child's medical notes and information collected during the study, held by the NHS, the Paediatric Intensive Care Audit Network or by NHS Digital, may be looked at by individuals from the sponsor organisation, the NHS Trust or the Intensive Care National Audit & Research Centre (ICNARC), where it is relevant to my child's taking part in this research. ___ ___ ___

4. I agree to my child's participation in the study and for all information collected about their hospital stay to be used as part of this research study ___ ___ ___

   OR

   I do <u>not</u> agree to my child's participation in the study and do not wish for any information collected about their hospital stay to be used as part of this research study ___ ___ ___

5. I agree for my child's samples to be analysed and stored as part of this research study ___ ___ ___

   OR

   I do <u>not</u> agree for my child's samples to be analysed and stored as part of this research study ___ ___ ___

6. I agree for my child's GP to be informed of their participation in the study ___ ___ ___

IRAS Number: 212228

7. I agree to be contacted about any future related studies ___ ___ ___

8. I agree that all information collected for the study, including samples, may be used to support other research in the future and may be shared anonymously with other researchers. ___ ___ ___

______________________	______________________	______________
Name of parent/guardian	Signature	Date (PRINT)
______________________	______________________	______________
Name of person taking consent	Signature	Date (PRINT)

Trial Number: ______________________________

Contact details (only needed if agreed to point 7)	
Telephone number: ______________________________	Mobile number: ______________________________
Email address: ____	
