# Supplementary material for: Protocol for a randomised pilot multiple centre trial of conservative versus liberal oxygenation targets in critically ill children (Oxy-PICU)
Source: BMJ Open. 2017 Dec 14;7(12):e019253. doi: 10.1136/bmjopen-2017-019253 (PMC5736024; doi:10.1136/bmjopen-2017-019253)
Supplement: Supplementary file 2 [file bmjopen-2017-019253supp002.pdf]

Trial Number ☐☐☐☐

Oxy-PICU

Parent/Guardian Consent Questionnaire

Directions

- The following questions are about the Oxy-PICU consent process that you took part in
- We refer to people agreeing to take part in research as ‘consenting’
- As your child received treatment as an emergency, consent for your child to take part in the Oxy-PICU research study would have been sought after the emergency situation
- This is known as research without prior consent, or deferred consent

Completing this questionnaire

Today’s date ☐☐/☐☐/☐☐☐☐

Are you the child’s ◯ Mother ◯ Father ◯ Other ________________ *(Please specify)*

Your child’s age ☐☐ days / weeks / months / years *(circle as appropriate)*

1. *Please indicate how strongly you agree or disagree with the following statements by placing a circle around the answer that best fits your opinion or decision*

**Statements**	**Agree**	**Neither agree nor disagree**	**Disagree**
a. The doctor or nurse checked that it was a convenient time to discuss research before discussing Oxy-PICU	1	2	3
b. I was initially surprised to find out that my child had already been entered into Oxy-PICU	1	2	3
c. The information I received about Oxy-PICU was clear and straightforward to understand	1	2	3
d. I understood why consent for my child's participation in Oxy-PICU was sought after the treatment had been given	1	2	3
e. I had enough opportunity to ask questions about Oxy-PICU	1	2	3
f. I was satisfied with the deferred consent process for Oxy-PICU	1	2	3
g. It was difficult to take in the information I was given about Oxy-PICU	1	2	3
h. It was difficult to make a decision about Oxy-PICU	1	2	3
i. I made this decision	1	2	3
j. Someone took this decision away from me	1	2	3
k. I was not in control of this decision	1	2	3
l. The decision about the research was inappropriately influenced by others	1	2	3

If the answer to this question is 'Agree', please state who you think influenced the decision about the research:

____

2. *Did you consent for your child to participate in Oxy-PICU?*

◯ Yes (Go to Question 3) ◯ No (Go to Question 4)

3. *What were your reasons for providing consent for your child to participate in Oxy-PICU?*

Please tick all that apply *<u>and then circle your main reason</u>* *(e.g. ✔)*

- a. To help my child ☐
- b. To help other children in the future ☐
- c. I felt that medical studies like Oxy-PICU are important ☐
- d. Because I trusted the doctor or nurse who explained Oxy-PICU ☐
- e. The treatment had already been given to my child ☐
- f. My child recovered ☐
- g. I didn't feel comfortable saying no to the nurse or doctor who explained the study ☐
- h. Other (Please state): ☐

____

4. *If you did not provide consent, please provide your reasons for deciding that your child would not take part in Oxy-PICU*
(If you do not wish to do so, please leave this space blank)

5. *We would value any comments or suggestions you have to improve the recruitment and consent process for Oxy-PICU*

We would like to thank you for taking the time to complete this questionnaire.

Please place the questionnaire in the envelope provided, seal it and give it back to the doctor or research nurse.
