# Supplementary material for: Protocol for a randomised pilot multiple centre trial of conservative versus liberal oxygenation targets in critically ill children (Oxy-PICU)
Source: BMJ Open. 2017 Dec 14;7(12):e019253. doi: 10.1136/bmjopen-2017-019253 (PMC5736024; doi:10.1136/bmjopen-2017-019253)
Supplement: Reviewer comments [file bmjopen-2017-019253.reviewer_comments.pdf]

PEER REVIEW HISTORY

BMJ Open publishes all reviews undertaken for accepted manuscripts. Reviewers are asked to complete a checklist review form (**http://bmjopen.bmj.com/site/about/resources/checklist.pdf**) and are provided with free text boxes to elaborate on their assessment. These free text comments are reproduced below.

ARTICLE DETAILS

**TITLE (PROVISIONAL)**	A protocol for a Randomised Pilot Multiple Centre Trial of Conservative versus Liberal Oxygenation Targets in Critically Ill Children (Oxy-PICU).
**AUTHORS**	Jones, Gareth; Ramnarayan, Padmanabhan; Raman, Sainath; Inwald, David; Grocott, Michael P. W.; Eaton, Simon; Ray, Samiran; Griksaitis, Michael; Pappachan, John; Wiley, Daisy; Mouncey, Paul; Wulff, Jerome; Harrison, David; Rowan, Kathryn; Peters, Mark

This article was not externally reviewed at BMJ Open. Protocols that have been independently assessed prior to submission to BMJ Open are usually fast-tracked to publication on the grounds that further substantial changes will not be possible. This independent assessment will usually be external, independent review for both a substantial grant award from a non-commercial or government funder and ethics approval.
